# Identification of Genetic Diagnostic Markers for Systemic Lupus Erythematosus

**DOI:** 10.1155/genr/7043184

**Published:** 2026-06-12

**Authors:** Qianqian Liu, Hairong Yang, Chunxiao Dang, Xingxing Song

**Affiliations:** ^1^ Shandong Provincial Hospital Affiliated to, Shandong First Medical University, Jinan, Shandong, China, sdfmu.edu.cn; ^2^ Dongying People’s Hospital (Dongying Hospital of Shandong Provincial Hospital Group), Dongying, Shandong, China; ^3^ Department of Acupuncture-Moxibustion and Tuina, Qilu Hospital of, Shandong University, Jinan, Shandong, China, sdu.edu.cn; ^4^ Department of Dermatology, Affiliated Hospital of Shandong University of Traditional Chinese Medicine, Jinan, Shandong, China, sdutcm.edu.cn

**Keywords:** bioinformatics, heredity, machine learning, systemic lupus erythematosus

## Abstract

**Background:**

Systemic lupus erythematosus (SLE) is a complex and heterogeneous systemic autoimmune disease associated with poor treatment outcomes. While previous studies have indicated a genetic predisposition to SLE, the underlying mechanisms remain poorly understood.

**Objective:**

This study aimed to identify diagnostic targets with potential genetic associations to SLE by leveraging bioinformatics and the Mendelian randomization (MR) approach.

**Methods:**

Six datasets (GSE30153, GSE39088, GSE50635, GSE50772, GSE61635, and GSE110169) were obtained from the GEO database for differential expression analysis to identify differentially expressed genes (DEGs). Weighted gene coexpression network analysis (WGCNA) was then performed, and the most relevant module was intersected with the DEGs to identify candidate genes with potential diagnostic value. Subsequently, machine learning algorithms were applied to screen diagnostic genes, and their performance was evaluated using receiver operating characteristic (ROC) curves and confusion matrices. MR analysis was conducted to identify diagnostic genes with genetic associations. A protein–protein interaction (PPI) network was constructed to identify core genes. Finally, gene set enrichment analysis (GSEA), gene set variation analysis (GSVA), and immune infiltration analysis were performed.

**Results:**

Differential expression analysis identified 244 DEGs, and WGCNA revealed a highly relevant module. Intersecting this module with the DEGs produced 136 candidate genes. Machine learning algorithms and MR analysis further refined the selection, identifying five diagnostic genes: GBP1, IFI6, KLHDC8B, OAS3, and ZCCHC2, all of which were shown to be well‐aligned with their respective drugs. The PPI network highlighted GBP1, IFI6, and OAS3 as core genes, which showed significant correlations with immune cell infiltration.

**Conclusions:**

Our study identified GBP1, IFI6, and OAS3 as core genes implicated in SLE pathogenesis, providing novel insights into its molecular mechanisms and potential therapeutic targets.

## 1. Introduction

Systemic lupus erythematosus (SLE) is a multiorgan chronic autoimmune disease with complex immune dysregulation and heterogeneous clinical manifestations [1]. It is characterized by a loss of immune tolerance, abnormal activation of autoreactive B and T cells, and the production of autoantibodies, which subsequently form immune complexes. These immune complexes can cause irreversible damage to multiple organs and systems in severe cases [2]. Studies have demonstrated a genetic predisposition to SLE [3, 4], with disease initiation involving abnormalities in multiple cellular signaling pathways and complex interactions between these pathways. The pronounced heterogeneity and intricate pathogenesis of SLE present significant challenges for accurate diagnosis and effective treatment. Traditional diagnostic approaches for SLE primarily rely on clinical and pathological indicators to evaluate disease severity; however, these methods often fail to uncover the underlying molecular mechanisms. Therefore, in‐depth investigation of the molecular pathogenic mechanisms of SLE is crucial, which not only helps us to diagnose the disease more accurately and determine the disease staging but also may provide key clues for the development of novel therapeutic targets.

In recent years, advancements in bioinformatics and machine learning have made their integration with the biomedical field indispensable. Studies have demonstrated that combining weighted gene coexpression network analysis (WGCNA) with machine learning models can provide deeper insights into the molecular mechanisms underlying SLE [5]. WGCNA facilitates the identification of coexpressed gene modules that may hold biological relevance to the disease [6], while machine learning techniques, such as random forest (RF) and least absolute shrinkage and selection operator (LASSO), can prioritize genes based on their diagnostic potential. This integrated approach not only enhances the robustness of biomarker discovery but also improves the accuracy and reliability of potential biomarkers for clinical application, thereby opening new avenues for the early diagnosis of SLE. Recent studies [7–9] have identified key genes involved in the pathogenesis of SLE using transcriptomic data analysis through similar bioinformatics methods, including differential expression analysis and machine learning. Despite these advances, it is important to recognize that SLE has a genetic predisposition. Previous studies have primarily focused on gene functions related to interferon responses, cytokine regulation, and immunomodulation, often neglecting the exploration of causal relationships between genes and SLE. Therefore, investigating the molecular features and mechanisms underlying the onset and progression of SLE from a genetic perspective is crucial for advancing early prevention, diagnosis, and treatment strategies for this disease.

Unlike previous studies that mainly focused on transcriptomic association analyses, our study integrated machine learning with WGCNA [10] and Mendelian randomization (MR) to identify diagnostic genes with both predictive value and potential genetic relevance, while further exploring their immune and therapeutic implications.

## 2. Material and Methods

### 2.1. Data Collection

Gene expression profiles related to SLE were obtained from the Gene Expression Omnibus (GEO) database (https://www.ncbi.nlm.nih.gov/geo/) using the search term “systemic lupus erythematosus.” The target organism was set to “*Homo sapiens*”, the entry type to “Series,” and the study type to “Expression profiling by array.” A total of 142 datasets were retrieved. To ensure data quality, we carefully examined the experimental sample information, experimental design, and data types within these datasets. Finally, six datasets were included in our study: GSE30153 (*n* = 26), GSE39088 (*n* = 124), GSE50635 (*n* = 49), GSE50772 (*n* = 81), GSE61635 (*n* = 109), and GSE110169 (*n* = 159). GSE61635 and GSE110169 were assigned to the training set because of their relatively larger sample sizes, which improved model training stability, while the remaining four datasets were used as independent external validation sets to evaluate model generalizability. Detailed information on all datasets is provided in Table 1. These six datasets were selected because they met the predefined inclusion criteria of *Homo sapiens*, series‐based microarray expression profiling, and availability of clearly annotated SLE and healthy control samples.

**TABLE 1:** Datasets and their characteristics.

Dataset	Platform	Sample
GSE50635	GPL6244	33 cases of SLE and 16 controls
GSE50772	GPL570	61 cases of SLE and 20 controls
GSE30153	GPL570	17 cases of SLE and 9 controls
GSE39088	GPL570	78 cases of SLE and 46 controls
GSE61635	GPL570	79 cases of SLE and 30 controls
GSE110169	GPL13667	82 cases of SLE and 77 controls

### 2.2. Screening for Differentially Expressed Genes (DEGs)

The training sets were combined and analyzed using the “SVA” package in *R* to account for batch effects. The data were normalized to eliminate these effects and ensure comparability across datasets. DEGs between 161 SLE samples and 107 healthy controls in GSE61635 and GSE110169 were identified using the “limma” package in *R* [11]. The *p*‐values were adjusted for multiple testing using the Benjamini–Hochberg method to control the false discovery rate (FDR). DEGs were selected based on an absolute fold‐change greater than 1.5 and a FDR‐adjusted *p*‐value (adj.P.Val) less than 0.05 as inclusion criteria. The expression levels of DEGs between SLE subjects and healthy controls were visualized using the “ggplot2″ and “heatmap” packages in *R*.

### 2.3. WGCNA Analysis

To identify gene modules associated with SLE, coexpression networks were constructed using the WGCNA package [12]. A soft‐thresholding power of 7 was selected to ensure the scale‐free topology of the network. Gene modules with similar expression profiles, each containing at least 60 genes (minModuleSize = 60), were identified using average linkage hierarchical clustering and the dynamic tree cut function based on the topological overlap measure (TOM) dissimilarity. Subsequently, module relationships were constructed, and correlations between gene modules and phenotypes were calculated using module eigengenes and module membership to identify key modules relevant to clinical traits. Finally, the modules with the highest Spearman correlation coefficients were identified as the significant modules.

### 2.4. Identification of Characteristic Genes and Enrichment Analysis

To systematically analyze gene functions and key biological pathways associated with SLE, we performed Gene Ontology (GO) enrichment analysis and Kyoto Encyclopedia of Genes and Genomes (KEGG) pathway analysis (https://www.genome.jp/kegg) [13] on the intersecting genes obtained in Section 2.3. The GO analysis results were categorized into three domains: biological processes (BPs), cellular components (CCs), and molecular functions (MFs) [14]. All analyses were conducted using the “clusterProfiler” package in *R*, and the *p*‐values were adjusted for multiple comparisons using the Benjamini–Hochberg (FDR) correction method. Statistical significance was defined as a FDR‐adjusted *p*‐value less than 0.05.

### 2.5. Construction and Evaluation of Machine Learning Diagnostic Model

We combined 12 machine learning algorithms to generate 113 algorithm combinations for further screening of the best diagnostic genes. These machine learning methods included RF [15], LASSO, ridge regression, elastic net (Enet), stepwise generalized linear model (Stepglm), support vector machine (SVM) [16], linear discriminant analysis (LDA), gradient boosting machine (GBM) [17], extreme gradient boosting machine (XGBoost) [18], naive Bayes (NB), generalized linear model boosting (glmBoost), and partial least squares regression generalized linear model (plsRglm). Supporting Excel 1 provided the advantages and disadvantages, detailed parameter settings, and the range of hyperparameter adjustments for 12 machine learning algorithms. GSE61635 and GSE110169 were combined as the training set, while GSE30153 and GSE39088 were used as validation sets. Fitted diagnostic models were constructed based on 10‐fold cross‐validation of the training set, and models producing fewer than five featured genes in the output were excluded. Receiver operating characteristic (ROC) curves were plotted for all validation datasets using the “pROC” package in *R* [19], and the area under the curve (AUC) was calculated to evaluate classification performance. The model with the highest average AUC value was considered the best‐performing model. Calibration curves were generated to assess the agreement between predicted and observed probabilities. Decision curve analysis was performed to evaluate the clinical net benefit of the model across a range of threshold probabilities. DeLong tests were used to compare the AUCs between the optimal model and competing models [20]. Finally, the accuracy and precision of the models were assessed using a confusion matrix. In addition, to improve the interpretability of the best‐performing machine learning model, Shapley additive explanations (SHAPs) were used as a supporting model‐level interpretation approach to visualize the cumulative contribution of multiple features to individual predictions [21].

### 2.6. MR Analysis and Evaluation of Common Genes

Candidate diagnostic genes identified from the optimal machine learning model in Section 2.5 were used as exposure factors, with SLE as the outcome factor. MR analyses were conducted using the “TwoSampleMR” software package [22] to identify diagnostic genes that are causally associated with SLE at the genetic level. Single nucleotide polymorphism (SNP) data were retrieved from publicly available eQTL databases, and SNPs with genome‐wide significance (*p* < 5 × 10^−8^) were selected as instrumental variables (IVs). SNPs that were significantly correlated with potential confounding factors and had an F‐statistic less than 10 were excluded. To ensure the independence of IVs, linkage disequilibrium (LD) pruning was performed (*R* ^2^ < 0.01, window size of 10,000 kb), and SNPs in significant LD were excluded. Exposure data were obtained from eQTL data in the publicly accessible IEU Open GWAS database (Supporting Excel 2, https://gwas.mrcieu.ac.uk/), while outcome data were sourced from the FinnGen database (https://r10.finngen.fi/pheno/SLE_OTH) (https://r10.finngen.fi/pheno/L12_LUPUS) (https://r10.finngen.fi/pheno/SDRUGADVERS_SYSTEMIC_LUPUS_ERYTHEMAT). The inverse variance weighted (IVW) method was used to evaluate the association between diagnostic genes and SLE risk. Additionally, MR Egger regression, weighted median (WME), simple mode (SM), and weighted mode (WM) methods were applied to supplement the IVW results. Heterogeneity and horizontal pleiotropy tests were performed to assess the validity of the IVs. Because multiple candidate genes were tested as exposures in the MR analyses, the *p*‐values from the primary MR estimates were further adjusted for multiple comparisons using the Benjamini–Hochberg FDR method. An FDR‐adjusted *p*‐value < 0.05 was considered statistically significant. Finally, ROC curves were plotted for each gene to evaluate their diagnostic ability.

### 2.7. Molecular Docking

Molecular docking was performed in order to identify potential drugs with good binding energy and interaction patterns with five causally linked diagnostic genes. This allowed us to optimize the design of potential drug candidates and select therapeutic targets with additional experimental validation. The PubChem compound database (https://pubchem.ncbi.nlm.nih.gov/) provides structural information on the drugs, and the protein database (http://www.rcsb.org/) provides protein structural information. The first two important drugs and 5 diagnostic genes were molecularly docked using CB‐Dock2 (https://cadd.labshare.cn/cb-dock2/index.php), and the results were presented using PyMOL 3.0.3 (https://www.pymol.org/). Finally, the final structures of 4 proteins and 2 drugs were successfully obtained.

### 2.8. Protein–Protein Interaction (PPI) Analysis

PPI networks were constructed using the GeneMANIA web platform to explore the interactions and functions of diagnostic genes. GeneMANIA utilizes GO‐based semantic similarity measurements to generate gene function predictions and identify genes with similar functions [23]. Thus, it was employed to evaluate the most critical genes among the diagnostic genes identified in the model.

### 2.9. GSEA and GSVA Enrichment Analysis

Global gene set enrichment analyses, including gene set enrichment analysis (GSEA) and gene set variation analysis (GSVA), were conducted to identify functionally coherent gene sets and differential activity in signal transduction cascades across the studied samples. The significance threshold for GSEA results was set at *p* < 0.05, and the analysis was visualized using the “enrichplot” package in R. GSVA was performed using the “GSVA” package [24] to quantify the enrichment of gene sets in each sample and evaluate the relative activity of gene sets across different samples.

### 2.10. Immune Infiltration Analysis

The CIBERSORT algorithm was applied to evaluate differences in immune cell infiltration between SLE and normal samples. The significance of these differences was assessed using the Wilcoxon test, with *p*‐values adjusted using the Benjamini–Hochberg FDR correction for comparisons across multiple immune cell types. The immune infiltration matrix was visualized with the “ggplot2” package in R. Specifically, CIBERSORT analysis was performed with 1000 permutations to robustly estimate the proportions of various immune cell types, applying a strict *p*‐value threshold of less than 0.05 to ensure that only significant differences in cellular composition were considered. Finally, the relationship between immune cells and diagnostic genes was analyzed using Spearman correlation analysis.

## 3. Results

### 3.1. Identify Shared Differential Genes

**FIGURE 1:**
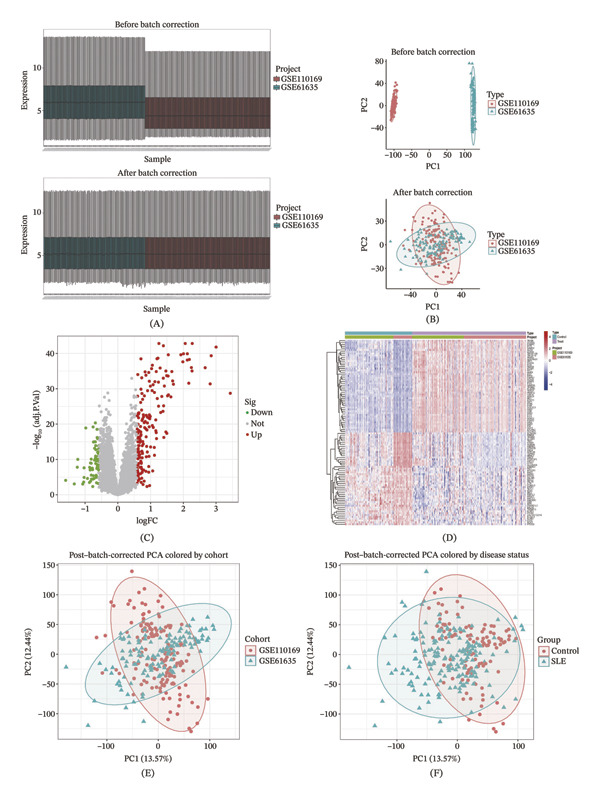
Identification of DEGs. (A) Box plots of gene expression before and after removal of batch effects. (B) PCA maps before and after the removal of batch effects. (C) Volcano map of DEGs. X‐axis: logFC, Y‐axis: ‐log10(adjusted *p*‐value); red, gray, and green dots represent upregulated, nonsignificant, and downregulated differentially expressed genes, respectively. (D) Heatmap of DEGs. Red and blue represent upregulated and downregulated genes, respectively. (E) PCA plot of the post–batch‐corrected training data colored by cohort. (F) PCA plot of the post–batch‐corrected training data colored by disease status.

After batch‐effect correction and normalization of GSE61635 and GSE110169, the merged training dataset showed improved comparability across samples (Figure 1A, B). Differential expression analysis identified 244 DEGs between SLE and healthy controls, including 179 upregulated and 65 downregulated genes (Figure 1C, D). These genes were used for subsequent network and feature‐selection analyses. To further assess the effectiveness of batch correction, PCA plots of the post‐batch‐corrected training data were generated with samples colored by cohort and disease status (Figure 1E‐F, Supporting Figure 1). These plots showed reduced cohort‐driven clustering after correction, while the separation between SLE and healthy controls remained partial.

### 3.2. Construction of WGCNA Network

**FIGURE 2:**
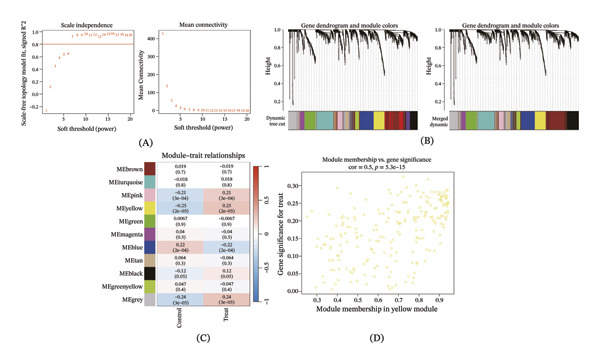
Identification of SLE‐associated key modules based on WGCNA analysis. (A) Scale‐free fitting index (left) and average connectivity (right) for different soft‐thresholding powers β. (B) Hierarchical clustering analysis was performed to detect coexpression clusters with specific color assignments, where each color represents a gene coexpression network module constructed by WGCNA. (C) Correlation heatmap showing the relationship between module eigengenes and SLE clinical traits. (D) The scatter plot in the yellow module illustrates the relationship between module membership (MM) and gene significance (GS).

WGCNA identified 11 coexpression modules in the training dataset. Using a soft‐thresholding power of 7, the network approximated a scale‐free topology (Figure 2A). Among the identified modules, the yellow module showed the highest correlation with SLE status (*r* = 0.25, *p* = 2 × 10^−5^), although the effect size was modest. Therefore, this module was retained as a candidate disease‐relevant module for downstream integrative analysis (Figure 2B–D).

### 3.3. GO and KEGG Enrichment Analysis

**FIGURE 3:**
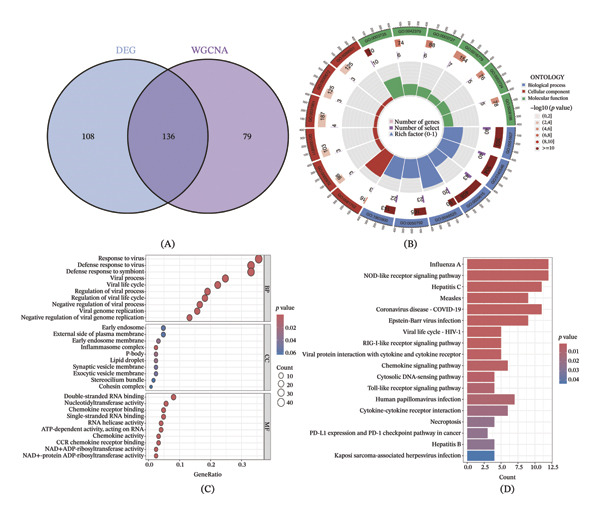
Intersection gene and functional enrichment analysis. (A) Venn diagram of 136 common genes between WGCNA and DEGs. (B) The circle diagram of GO enrichment analysis. (C) Bubble map of GO enrichment analysis. (D) Histogram of KEGG enrichment analysis.

Intersection of the DEGs with genes from the yellow module yielded 136 candidate genes (Figure 3A). Functional enrichment analysis indicated that these genes were mainly involved in antiviral defense and immune response‐related processes. Representative GO terms included defense response to virus, inflammasome complex, and double‐stranded RNA binding (Figure 3B,C). KEGG analysis further showed enrichment in the NOD‐like receptor signaling pathway, RIG‐I‐like receptor signaling pathway, and chemokine signaling pathway (Figure 3D, Supporting Excels 3–4), suggesting that these candidate genes are closely linked to innate immune activation in SLE.

### 3.4. Establishment and Validation of Machine Learning Model

**FIGURE 4:**
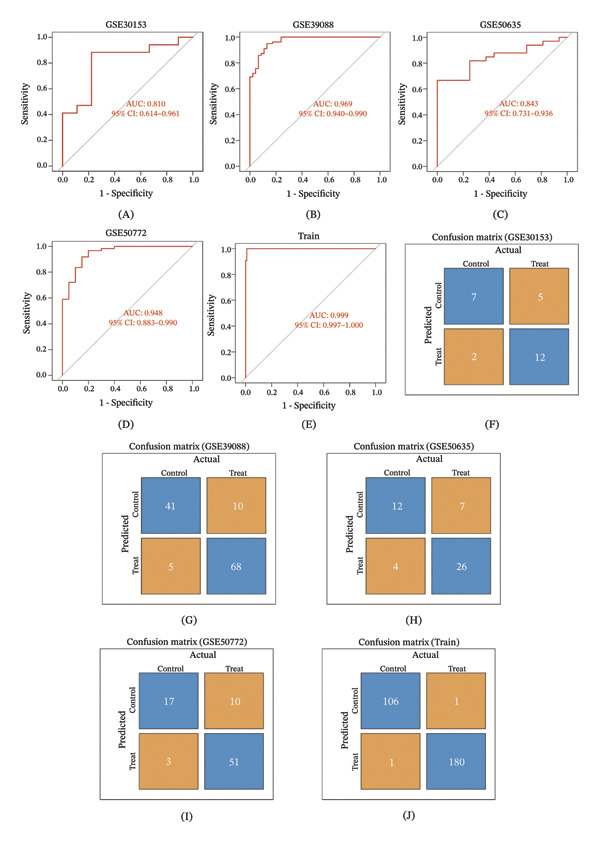
Machine learning algorithm analysis. (A–E) ROC curves for the training and validation sets. The *x*‐axis represents 1‐specificity, and the *y*‐axis represents sensitivity. The area under the curve was used to evaluate the diagnostic performance of the model. (F–J) Confusion matrices of the optimal machine learning model in the training set and external validation datasets, showing the classification results for SLE and healthy control samples.

To identify diagnostic genes associated with SLE, the 136 overlapping genes between WGCNA and DEGs were analyzed using multiple machine‐learning algorithms, including RF, LASSO, ridge regression, Enet, Stepglm, SVM, GBM, NB, and XGBoost. Among these, the GBM model achieved the best performance, with the highest AUC (0.914) (Supporting Figure 2). Its diagnostic value was further supported in external validation datasets, with AUCs of 0.810, 0.969, 0.843, and 0.948 in GSE30153, GSE39088, GSE50635, and GSE50772, respectively. Notably, the relatively lower AUC observed in GSE30153 may be partly attributable to differences in sample type, as this dataset consists of isolated B cells, whereas the training model was developed using whole blood samples. The nearly perfect performance in the training set indicates the possibility of potential overfitting. Therefore, the validation‐set performance is likely more informative for judging the generalizability of the model (Figure 4A–E). Calibration curves and decision curve analysis indicated favorable model performance across cohorts, while DeLong testing showed no significant difference in AUC compared with other leading models, despite numerically better performance in some validation sets (Supporting Excel 5, Supporting Figure 3). Confusion matrix analysis further supported the classification ability of the GBM model (Figure 4F–J). SHAP analysis indicated that predictions were influenced by multiple features rather than a single dominant variable (Supporting Figure 4).

### 3.5. MR Analysis Results and Evaluation

**FIGURE 5:**
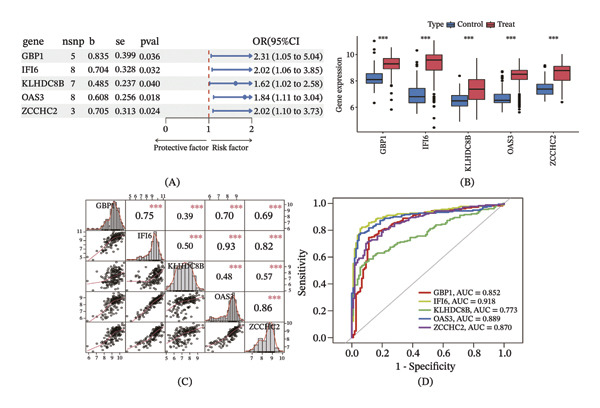
Identification and differential expression of diagnostic genes. (A) Forrest plot for a causal association between diagnostic genes and SLE. The effect size is shown as the odds ratio, and horizontal lines indicate the corresponding 95% confidence intervals. (B, C) Differential expression of diagnostic genes in the normal population and SLE patients. (D) ROC curves of diagnostic genes. The area under the curve indicating classification ability.

MR analysis was performed using genes from the optimal GBM model as exposures and SLE as the outcome. Five genes—GBP1, IFI6, KLHDC8B, OAS3, and ZCCHC2—showed significant positive genetic associations with SLE (Figure 5A). After FDR correction, all five genes remained statistically significant (Supporting Excel 6). Sensitivity analyses showed no evidence of horizontal pleiotropy, and leave‐one‐out analysis supported the robustness of the findings, although IFI6 showed heterogeneity and was therefore interpreted primarily according to the WME estimate (Supporting Excel 7, Supporting Figure 5). All five genes also demonstrated diagnostic potential, with AUC values above 0.7, among which IFI6, OAS3, and GBP1 showed the best performance (Figure 5D). Based on the combined MR and diagnostic analyses, these genes were prioritized for downstream evaluation. MR results for all 136 candidate genes are provided in Supporting Excel 8.

### 3.6. Molecular Docking

**FIGURE 6:**
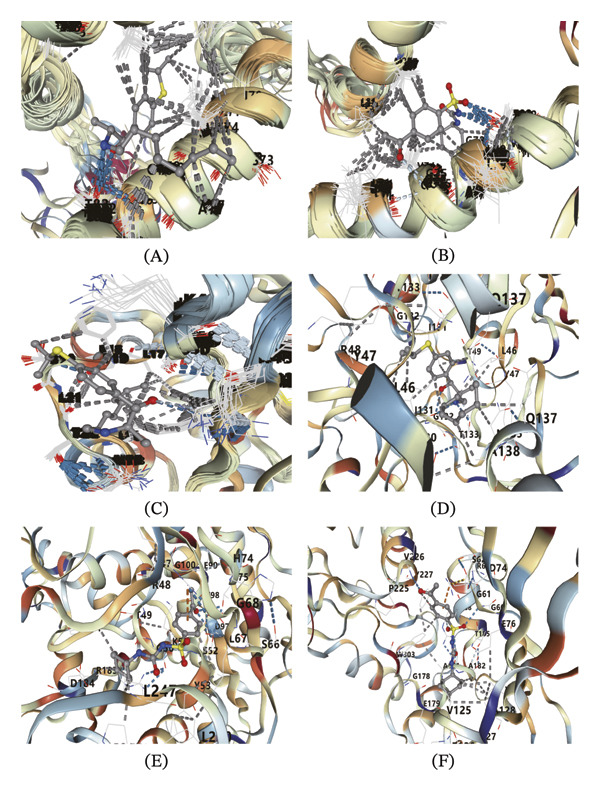
Molecular docking results of available proteins and drugs. (A) IFI6 docking suloctidil, (B) IFI6 docking 3′‐azido‐3′‐deoxythymidine, (C) ZCCHC2 docking suloctidil, (D) GBP1 docking suloctidil, (E) GBP1 docking 3′‐azido‐3′‐deoxythymidine, (F) OAS3 docking 3′‐azido‐3′‐deoxythymidine.

Molecular docking suggested favorable binding between several candidate drugs and proteins encoded by IFI6, ZCCHC2, GBP1, and OAS3, providing preliminary support for their potential therapeutic relevance (Figure 6, Supporting Excel 9).

### 3.7. PPI Network

**FIGURE 7:**
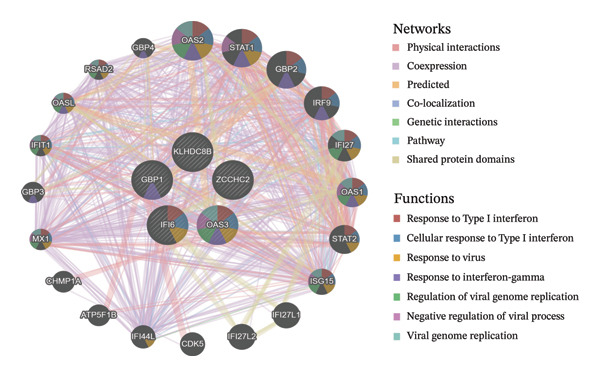
PPI network of diagnostic genes.

PPI network analysis highlighted OAS3, IFI6, and GBP1 as the most central genes among the five genetically associated diagnostic markers, and these three genes were therefore considered the core genes of the study (Figure 7).

### 3.8. GSEA and GSVA

**FIGURE 8:**
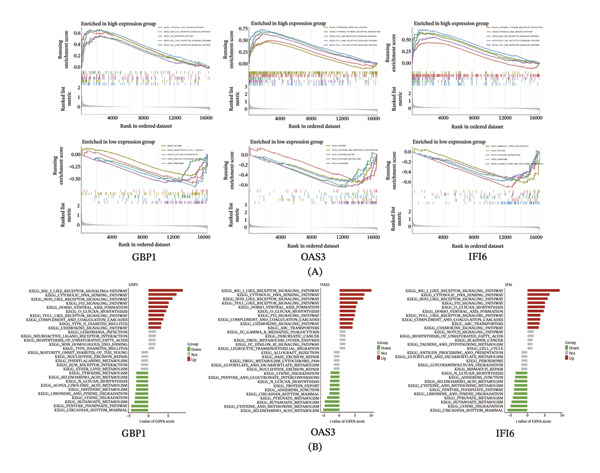
Functional analysis of core genes. (A) GSEA analysis of GBP1, IFI6, and OAS3. The plots show pathways enriched in the high‐ and low‐expression groups of the core genes, highlighting the major biological processes associated with their expression patterns. (B) GSVA of GBP1, IFI6, and OAS3, showing pathway activity differences associated with the expression levels of the core genes across samples.

GSEA was conducted to identify functional alterations in DEGs between normal populations and SLE patients. KEGG analysis of the high‐expression group revealed that the core genes were primarily involved in the RIG‐I‐like receptor signaling pathway. In contrast, KEGG analysis of the low‐expression group indicated that the core genes were mainly associated with the ribosome (Figure 8A).

GSVA was conducted to identify functional alterations in each DEG. In the KEGG analysis, the high‐expression group showed that the functions of the core genes were primarily involved in the RIG‐I‐like receptor signaling pathway. Conversely, in the low‐expression group, KEGG analysis indicated that the enrichment of OAS3 was mainly associated with selenoamino acid metabolism, while IFI6 and GBP1 were primarily involved in the circadian rhythm mammal pathway (Figure 8B).

### 3.9. Immune Landscape Characterization

**FIGURE 9:**
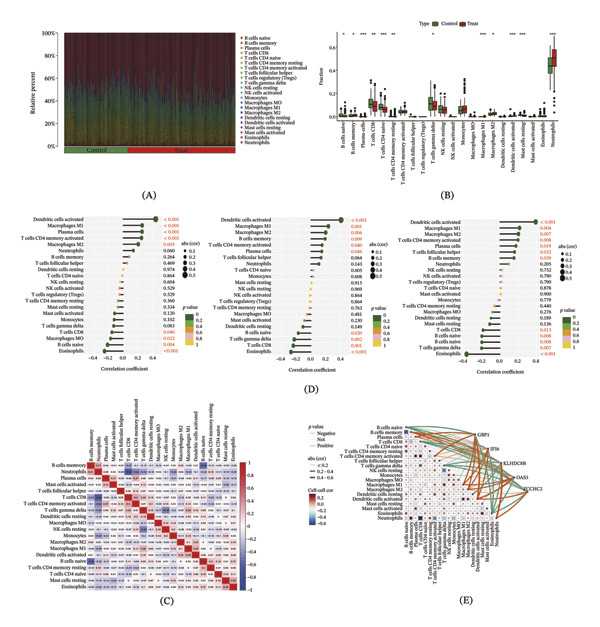
Immune landscape characterization in SLE and healthy controls. (A) Heatmap showing the relative abundance of infiltrating immune cells across samples. (B) Comparison of immune cell proportions between SLE and healthy control groups. (C) Correlation heatmap showing the relationships among different immune cell subsets. (D) Correlation analysis between the core genes (OAS3, IFI6, and GBP1) and immune cell infiltration levels. (E) Summary of correlation coefficients between the core genes and immune cell subsets.

Immune infiltration analysis revealed significant differences in several immune cell populations between SLE and controls, including plasma cells, naive CD4 T cells, macrophages, activated dendritic cells (DCs), and neutrophils (Figure 9). GBP1, IFI6, and OAS3 were broadly correlated with multiple immune cell subsets, further supporting their potential involvement in immune dysregulation in SLE.

## 4. Discussion

SLE is a prevalent autoimmune disease [25], but its heterogeneity poses significant challenges for both diagnosis and treatment due to its diverse clinical manifestations and lack of specific biomarkers [26]. The diagnostic criteria for SLE are based on a combination of clinical features, such as joint pain and psychoneurological symptoms, as well as laboratory tests, including antinuclear antibodies (ANAs). However, these criteria often suffer from poor specificity and sensitivity, leading to ongoing controversy regarding their clinical application. Increasing evidence suggests that SLE is a hereditary autoimmune disease involving multiple genes, as demonstrated by various studies [26, 27]. In this study, after identifying diagnostic genes using WGCNA and machine learning algorithms, we further screened for diagnostic genes with genetic associations through MR analysis. This approach enabled us not only to identify genes with predictive value but also to further assess their potential genetic relevance to SLE. Moreover, through functional enrichment analysis, immune infiltration analysis, PPI network construction, and molecular docking, we explored the biological functions, immune‐associated mechanisms, and possible therapeutic significance of the identified genes. Therefore, our study extends previous work by providing a more integrative framework for understanding SLE pathogenesis and for prioritizing candidate biomarkers and potential therapeutic targets.

Our integrative analysis prioritized GBP1, IFI6, and OAS3 as core candidate genes associated with SLE. Functionally, these genes were enriched in interferon‐ and innate immune‐related pathways, supporting their biological relevance to the inflammatory and antiviral signatures that characterize SLE. In SLE patients, increased secretion of IFN‐γ activates the transcription of Class I and Class II MHC molecules, thereby exacerbating the disease [28]. GBP1, a key interferon‐stimulated gene, is overexpressed in response to IFN‐γ activation, and its products inhibit cell proliferation and apoptosis during early cellular inflammatory responses [29, 30]. Although limited research has been conducted on the relationship between GBP1 and SLE, previous studies [31] have highlighted the important role of GBP1 and IFN‐γ in the early onset of SLE. Further investigation into the role of GBP1 in SLE is warranted. Similarly, IFI6, another interferon‐stimulated gene, has been reported to play an important role in rheumatoid arthritis [32], but relatively few studies have explored its role in SLE. In 2016, Bing PF et al. [33] proposed IFI6 as a new marker gene for SLE, but there is still no definitive answer as to whether or not IFI6 can be used in the diagnosis of SLE. 2′‐5′‐Oligoadenylate synthase 3 (OAS3) is a class of interferon‐induced antiviral proteins that synthesize secondary messengers in response to viral infections, inducing RNA decay in infected cells and effectively inhibiting further viral replication [34]. A study [35] demonstrated that OAS3 expression is upregulated in CD4 T cells, CD19 B cells, and CD33 myeloid cells from SLE patients, suggesting its involvement in the pathogenesis of SLE by mediating the proinflammatory effects of IFN‐α‐2a. Additionally, Grammatikos et al. [36] proposed that the expression level of OAS3 in T cells could be used to diagnose and monitor disease activity in SLE patients.

SLE diagnosis in clinical practice still depends mainly on established laboratory indicators, including ANA, anti‐dsDNA antibodies, anti‐Sm antibodies, and complement consumption, all of which have recognized limitations in sensitivity, specificity, or disease‐stage applicability [37, 38]. In addition, several interferon‐stimulated genes, such as IFIT1 and MX1, have been reported as SLE‐associated biomarkers and are considered part of the interferon signature of the disease [39]. Similar to these known markers, GBP1, IFI6, and OAS3 are closely linked to interferon‐driven innate immune activation, supporting their biological relevance in SLE. However, the novelty of the present study lies not merely in identifying additional interferon‐related genes but in applying an integrative framework that combines differential expression analysis, WGCNA, machine learning, and MR to prioritize candidate biomarkers with both diagnostic relevance and genetic support for SLE risk. Therefore, these genes may complement rather than replace conventional biomarkers and may add etiological support to the established interferon‐signature biomarker landscape. Nevertheless, GBP1, IFI6, and OAS3 should currently be regarded as candidate biomarkers rather than clinically validated diagnostic markers. Further studies are needed to validate them at the mRNA and, where feasible, protein levels, to compare their incremental value against existing laboratory tests, and to establish clinically meaningful cutoff values and reproducibility in prospective multicenter cohorts.

As an autoimmune disease, SLE is strongly influenced by innate immune cells, which play an important role in its pathogenesis. Monocytes can differentiate peripherally into macrophages and DCs. Recently, it has been shown that plasmacytoid dendritic cells (pDC) from SLE patients produce large amounts of IFN‐α, which upon binding to receptors activate the JAK–STAT signaling pathway and positive feedback stimulates the activation of pDC and T cells [40]. In addition, an imbalance between macrophage polarization and aberrant activation likewise underlies the development of SLE [41, 42]. In SLE, neutrophil activation, increased aggregation, enhanced apoptotic tendency, impaired phagocytosis, increased mitochondrial ROS production, and an increased propensity for spontaneous release of NET are observed [43–45]. NET contributes to the pathogenesis of SLE through various mechanisms, such as exposure to autoantigens and activation of autoreactive B cells [45–47]. We used the CIBERSORT algorithm to analyze and compare the abundance of immune cell infiltration in the SLE group with that in the healthy group, and the results showed the presence of a number of immune cells, including “B cells naive,” “Plasma cells,” “T cells CD4 naive,” “Macrophages,” “Dendritic cells activated,” and “Neutrophils,” with statistically significant differences between the two groups. GBP1, OAS3, and IFI6 were significantly correlated with a wide range of immune cells. Therefore, analyzing the association between these diagnostic genes and immune infiltration in SLE patients may provide new targets and strategies to attenuate the immune dysregulation response in SLE.

While our study provides valuable insights, there are several limitations. First, the included GEO datasets were derived from public databases, detailed demographic and clinical characteristics were not consistently available across studies; therefore, strict matching across datasets could not be performed, which may have introduced residual heterogeneity. Second, the dataset used in this study was generated using microarray technology, and inconsistencies between different assay platforms and variability due to differing sample sources may affect the reliability and generalizability of our findings. In addition, although multiple independent cohorts were included in this study, validation in larger and more diverse public lupus transcriptomic datasets would further strengthen the robustness and generalizability of the identified gene signature. Third, CIBERSORT analyses are based on limited genetic data, which may not fully account for cellular heterotypic interactions, disease‐induced disorders, or phenotypic plasticity. In addition, most of the identified genes are involved in interferon and antiviral response pathways, which may also be activated in other autoimmune, inflammatory, or infectious conditions; therefore, their disease specificity for SLE remains to be further established. Most importantly, the present study lacks direct experimental validation. Therefore, the identified genes should be considered candidate biomarkers supported by integrative bioinformatics and MR evidence. Further experimental studies, including validation in independent clinical samples and functional assays *in vitro* and *in vivo*, are needed to confirm their biological functions and diagnostic value in SLE.

## 5. Conclusions

Our study identified GBP1, IFI6, and OAS3 as core candidate genes associated with SLE and highlighted their potential relevance as transcriptome‐based biomarkers. These findings provide further insight into the molecular mechanisms of SLE and may support future biomarker development. However, prospective clinical validation, assay standardization, and functional experiments are still required before clinical application.

**Nomenclature**
SLE: systemic lupus erythematosus
MR: Mendelian randomization
DEGs: differentially expressed genes
WGCNA: weighted gene co‐expression network analysis
ROC: receiver operating characteristic
PPI: protein–protein interaction
GSEA: gene set expression analysis
GSVA: gene set variation analysis
SNPs: single nucleotide polymorphisms
GO: gene ontology
KEGG: Kyoto Encyclopedia of Genes and Genomes
BP: biological process
CC: cellular component
MF: molecular function
IVW: inverse variance weighted
WME: weighted median
SM: simple mode
WM: weighted mode

## Author Contributions

Qianqian Liu: data curation, visualization, and writing–original draft. Hairong Yang: data curation, formal analysis, and writing–original draft. Chunxiao Dang: conceptualization, software, and visualization. Xingxing Song: resources, supervision, formal analysis, and writing–review and editing.

## Funding

No funding was received for this research.

## Ethics Statement

The manuscript does not contain clinical studies or patient data.

## Consent

The authors have nothing to report.

## Conflicts of Interest

The authors declare no conflicts of interest.

## Supporting Information

Additional supporting information can be found online in the Supporting Information section.

## Supporting information


**Supporting Information** Supporting Excel 1. Commonly used parameter settings for the 12 machine learning algorithms and their advantages and disadvantages. Supporting Excel 2. eQTL data. Supporting Excel 3. GO functional analysis of intersection genes in biological processes (BPs), cellular components (CCs), and molecular functions (MFs). Supporting Excel 4. KEGG pathway enrichment analysis of intersection genes. Supporting Excel 5. Statistical comparison of ROC AUCs using the DeLong test. Supporting Excel 6. MR analysis results of hub genes on SLE. Supporting Excel 7. Heterogeneity and pleiotropy test results of hub genes and SLE. Supporting Excel 8. MR analysis results of all genes. Supporting Excel 9. DRUG enrich. Supporting Figure 1. Principal component analysis (PCA) of the post‐batch‐corrected training data displayed by cohort and disease status. Each point represents one sample; colors indicate cohort, and shapes indicate disease status. This combined view was provided to further visualize the distribution of SLE and control samples across datasets after batch‐effect correction. Supporting Figure 2. Construction of 113 machine learning models. Supporting Figure 3. Calibration performance and clinical utility of the optimal GBM model assessed by calibration curves and decision curve analysis. (A‐E) Calibration curves. (F‐J) Decision curves. Supporting Figure 4. SHAP‐based interpretation of the optimal GBM model. (A) The top 19 highly predictive features in the GBM model. (B‐C) Representative waterfall plots are shown to illustrate the cumulative contribution of multiple features to individual predictions. Supporting Figure 5. Results of leave‐one‐out analysis of core genes causally associated with SLE. (A) GBP1, (B) IFI6, (C) KLHDC8B, (D) OAS3, (E) ZCCHC2.

## Data Availability

Data supporting the findings of this study are available from the paper and its Supporting Information document.
