# Supplementary material for: Identification of Genetic Diagnostic Markers for Systemic Lupus Erythematosus
Source: Genet Res (Camb). 2026 Jun 12;2026:7043184. doi: 10.1155/genr/7043184 (PMC13263162; doi:10.1155/genr/7043184)
Supplement: Supplementary file 1 — Supporting Information Supporting Excel 1. Commonly used parameter settings for the 12 machine learning algorithms and their advantages and disadvantages. Supporting Excel 2. eQTL data. Supporting Excel 3. GO functional analysis of intersection genes in biological processes (BPs), cellular components (CCs), and molecular functions (MFs). Supporting Excel 4. KEGG pathway enrichment analysis of intersection genes. Supporting Excel 5. Statistical comparison of ROC AUCs using the DeLong test. Supporting Excel 6. MR analysis results of hub genes on SLE. Supporting Excel 7. Heterogeneity and pleiotropy test results of hub genes and SLE. Supporting Excel 8. MR analysis results of all genes. Supporting Excel 9. DRUG enrich. Supporting Figure 1. Principal component analysis (PCA) of the post‐batch‐corrected training data displayed by cohort and disease status. Each point represents one sample; colors indicate cohort, and shapes indicate disease status. This combined view was provided to further visualize the distribution of SLE and control samples across datasets after batch‐effect correction. Supporting Figure 2. Construction of 113 machine learning models. Supporting Figure 3. Calibration performance and clinical utility of the optimal GBM model assessed by calibration curves and decision curve analysis. (A‐E) Calibration curves. (F‐J) Decision curves. Supporting Figure 4. SHAP‐based interpretation of the optimal GBM model. (A) The top 19 highly predictive features in the GBM model. (B‐C) Representative waterfall plots are shown to illustrate the cumulative contribution of multiple features to individual predictions. Supporting Figure 5. Results of leave‐one‐out analysis of core genes causally associated with SLE. (A) GBP1, (B) IFI6, (C) KLHDC8B, (D) OAS3, (E) ZCCHC2. [file GENR-2026-7043184-s001.zip › Supplementary Figures.docx]

**
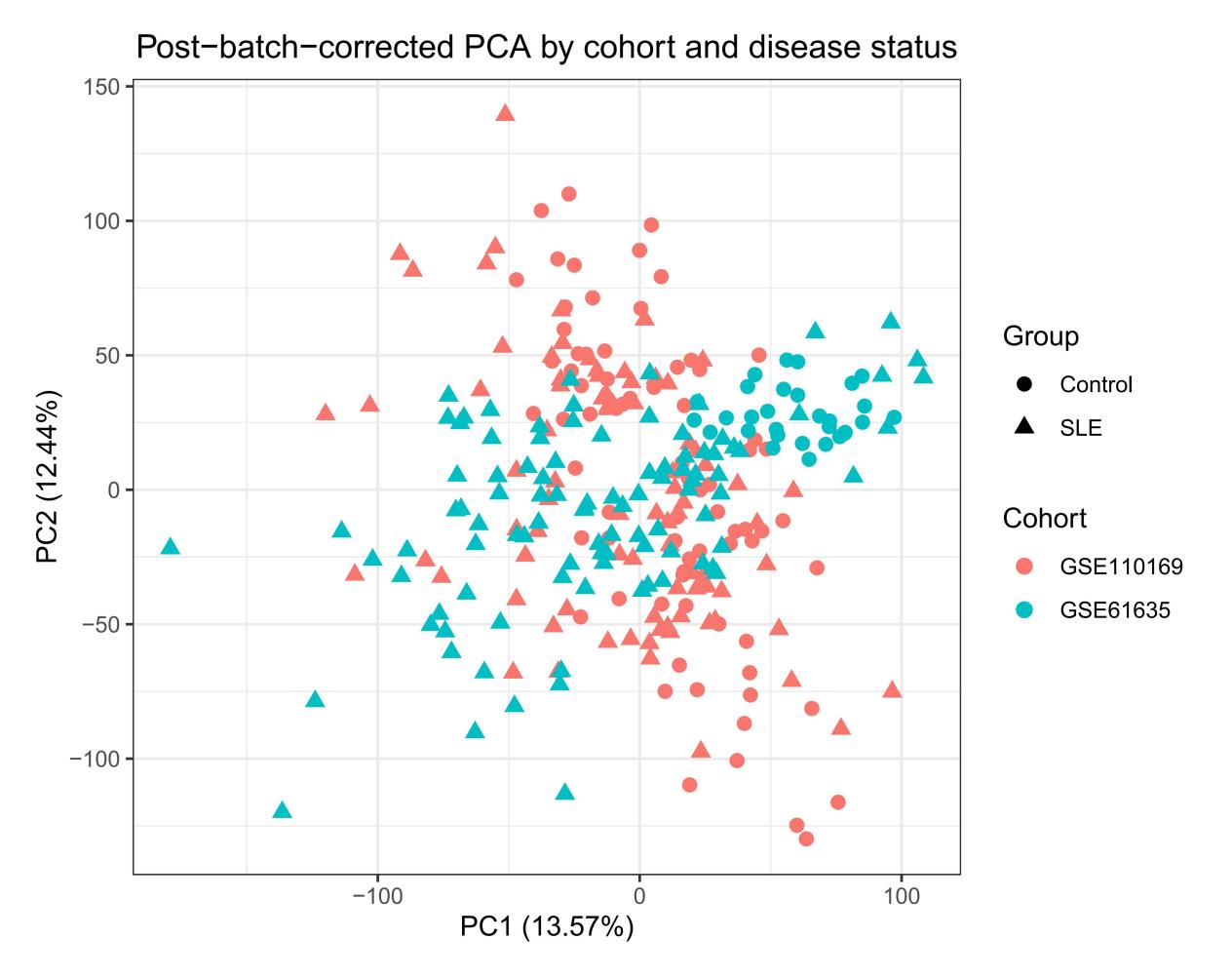
**

**Supplementary Figure 1.** Principal component analysis (PCA) of the post-batch-corrected training data displayed by cohort and disease status. Each point represents one sample; colors indicate cohort and shapes indicate disease status. This combined view was provided to further visualize the distribution of SLE and control samples across datasets after batch-effect correction.


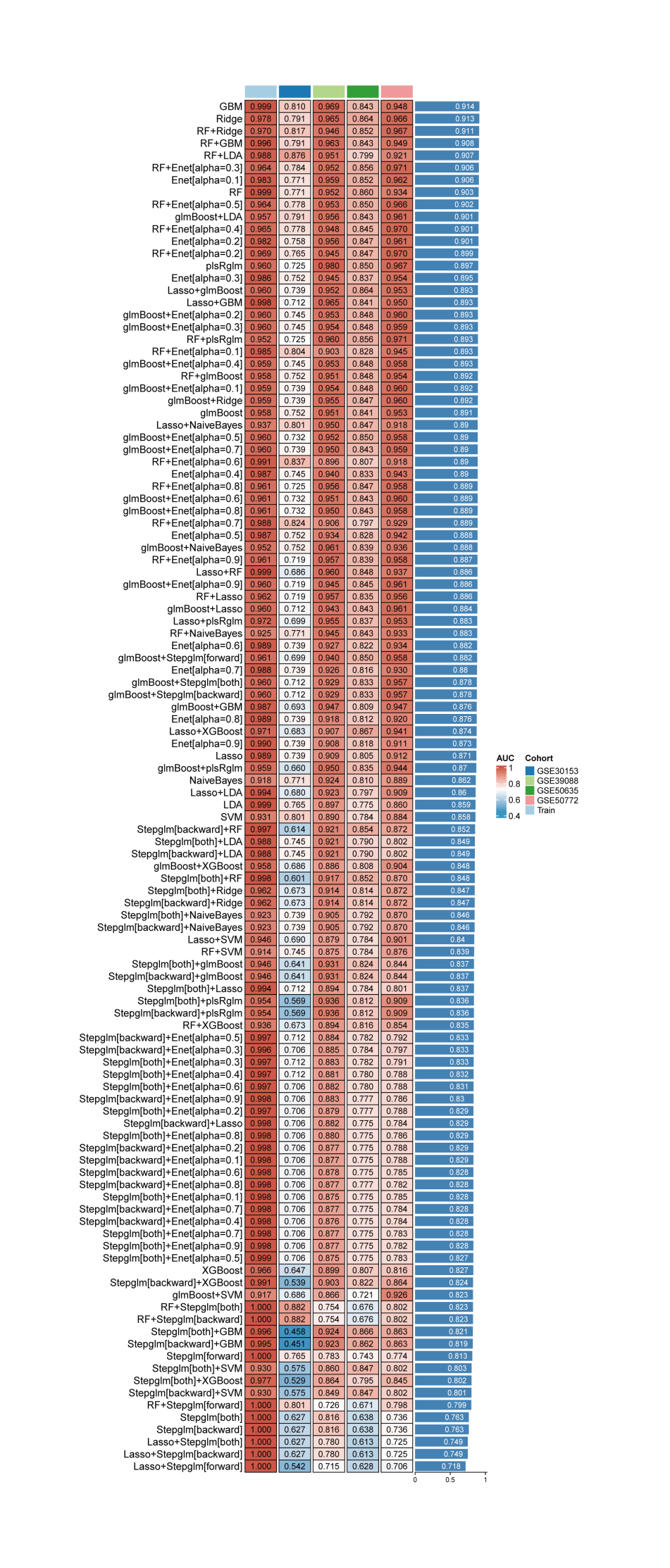


**Supplementary Figure 2.** Construction of 113 machine learning models.


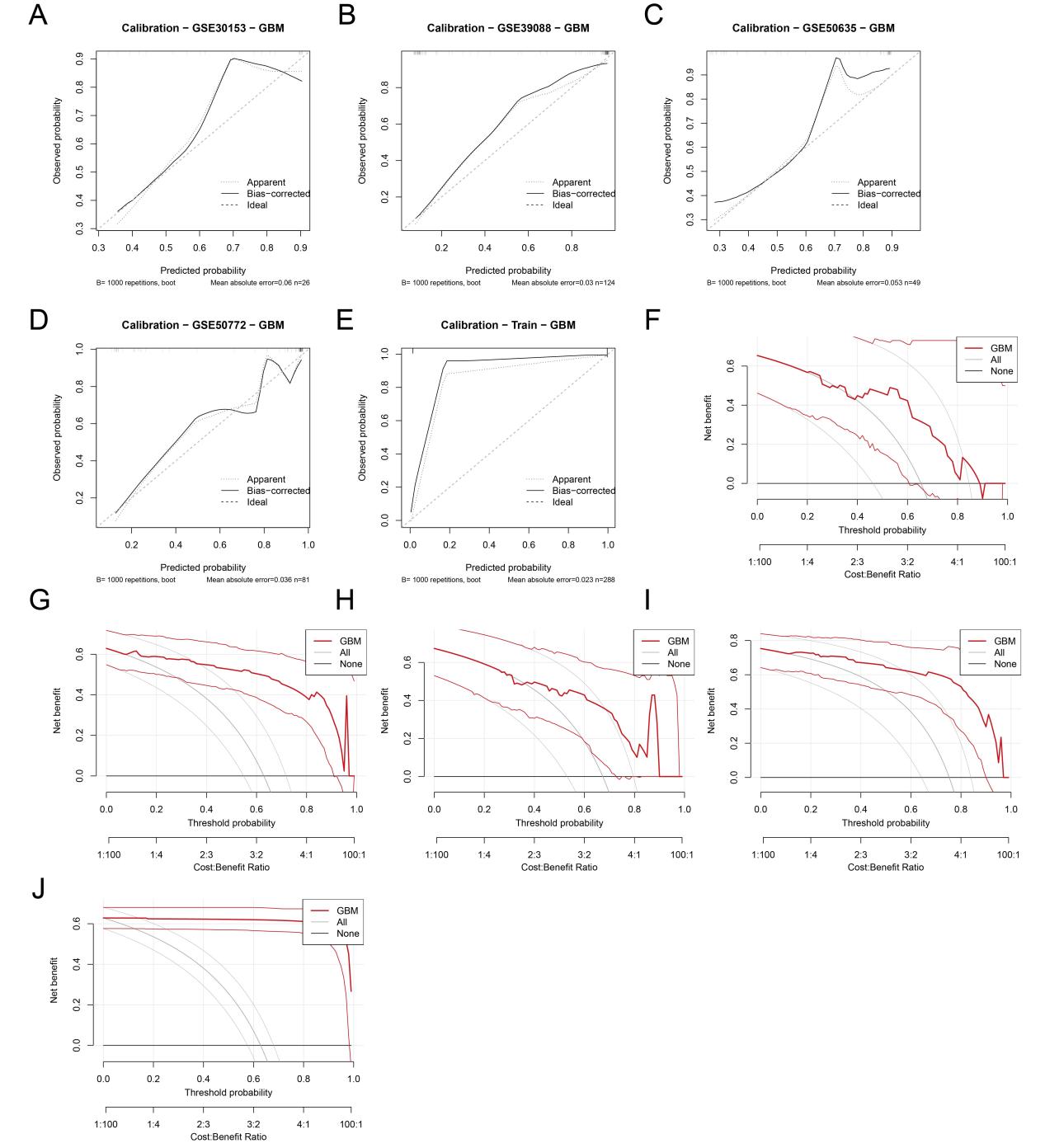


**Supplementary Figure 3.** Calibration performance and clinical utility of the optimal GBM model assessed by calibration curves and decision curve analysis. (A-E) Calibration curves. (F-J) Decision curves.


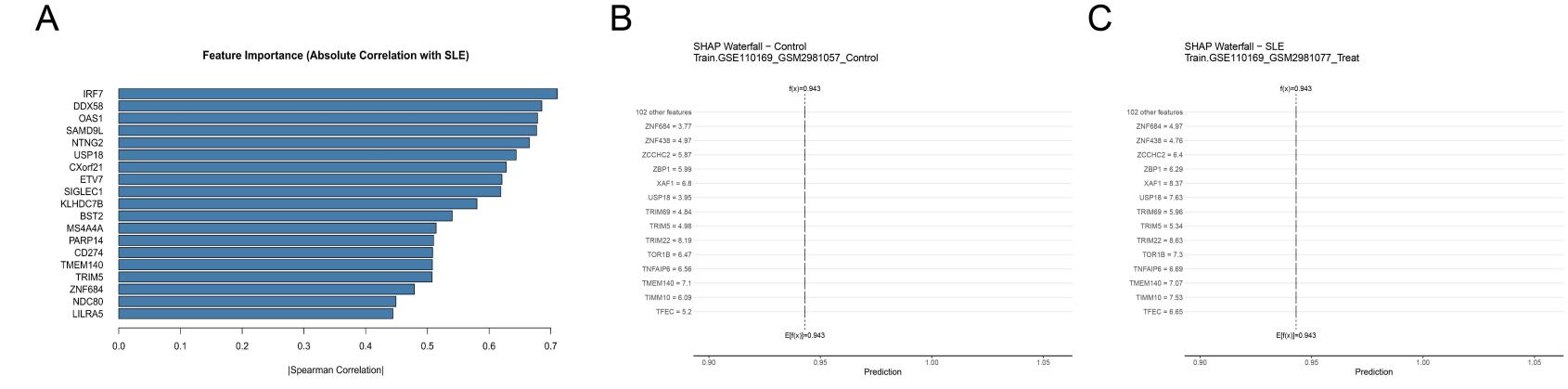


**Supplementary Figure 4.** SHAP-based interpretation of the optimal GBM model. (A) The top 19 highly predictive features in the GBM model. (B-C) Representative waterfall plot are shown to illustrate the cumulative contribution of multiple features to individual predictions.


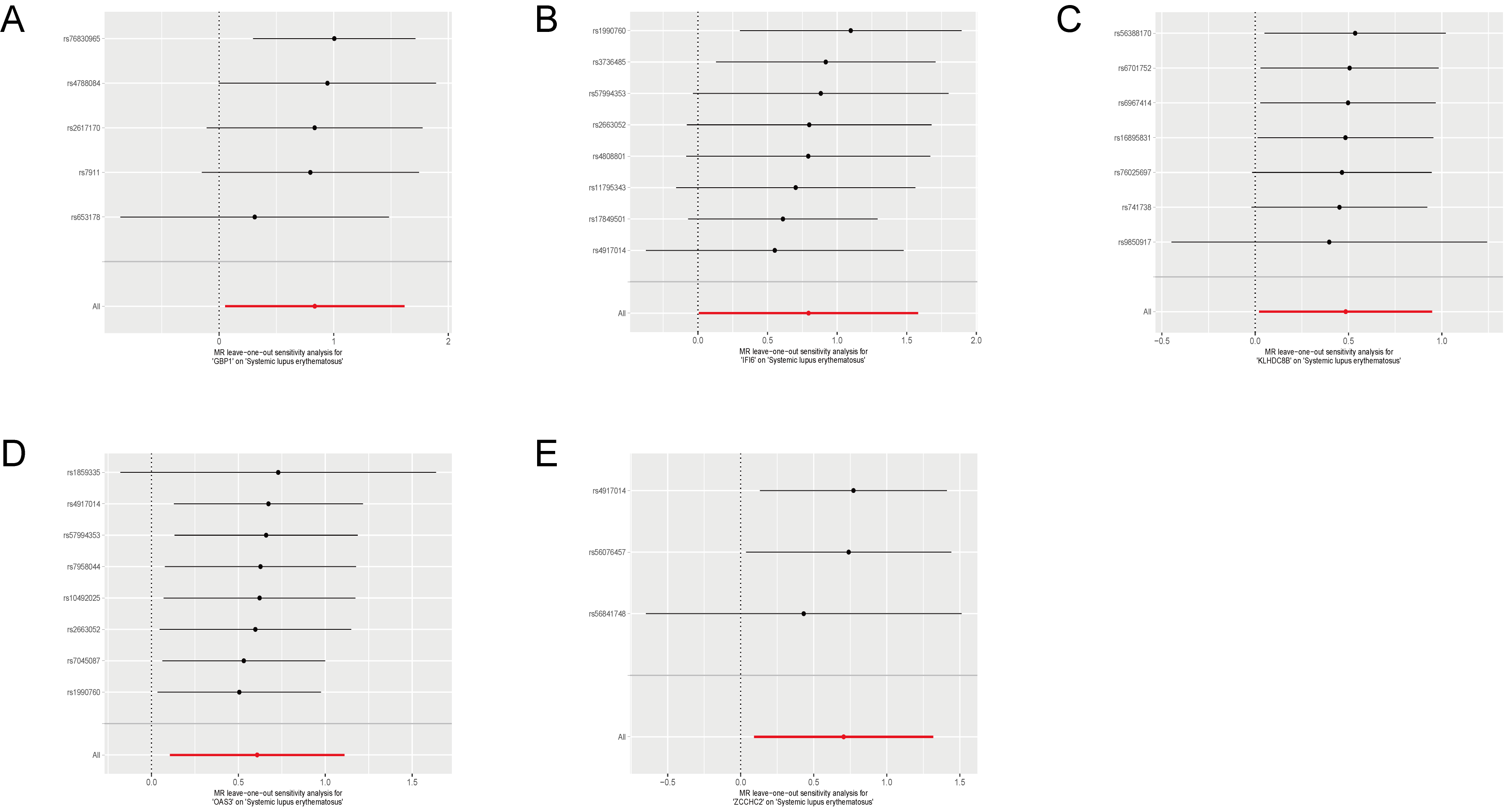


**Supplementary Figure 5.** Results of Leave-one-out analysis of core genes causally associated with SLE. (A) GBP1 (B) IFI6 (C) KLHDC8B (D) OAS3 (E) ZCCHC2.
